# Neuroenhancement of Exposure Therapy in Anxiety Disorders

**DOI:** 10.3934/Neuroscience.2015.3.123

**Published:** 2015-07-24

**Authors:** Stefan G. Hofmann, Elizabeth A. Mundy, Joshua Curtiss

**Affiliations:** Psychotherapy and Emotion Research Laboratory, Department of Psychological and Brain Sciences, Boston University, Boston, MA USA

**Keywords:** neuroenhancer, cognitive enhancer, exposure therapy, extinction, d-cycloserine, cognitive behavioral therapy, anxiety disorders

## Abstract

Although exposure-based treatments and anxiolytic medications are more effective than placebo for treating anxiety disorders, there is still considerable room for further improvement. Interestingly, combining these two modalities is usually not more effective than the monotherapies. Recent translational research has identified a number of novel approaches for treating anxiety disorders using agents that serve as neuroenhancers (also known as cognitive enhancers). Several of these agents have been studied to determine their efficacy at improving treatment outcome for patients with anxiety and other psychiatric disorders. In this review, we examine d-cycloserine, yohimbine, cortisol, catecholamines, oxytocin, modafinil, and nutrients such as caffeine and amino fatty acids as potential neuroenhancers. Of these agents, d-cycloserine shows the most promise as an effective neuroenhancer for extinction learning and exposure therapy. Yet, the optimal dosing and dose timing for drug administration remains uncertain. There is partial support for cortisol, catecholamines, yohimbine and oxytocin for improving extinction learning and exposure therapy. There is less evidence to indicate that modafinil and nutrients such as caffeine and amino fatty acids are effective neuroenhancers. More research is needed to determine their long term efficacy and clinical utility of these agents.

## 1. Introduction

Behavioral and exposure based treatments, such as cognitive behavioral therapy (CBT), are among the most efficacious for anxiety disorders [1,2]. The success of these interventions is, in part, a consequence of their targeting core mechanisms implicated in the genesis and maintenance of pathological anxiety: maladaptive learning and fear conditioning. A standard course of exposure based treatment entails exposure to feared objects or situations and the elimination of safety behaviors (i.e., subtle avoidance behaviors that temporarily diminish distress in feared situations, but fail to result in long-term reductions in anxiety). Patients are encouraged to encounter feared objects without engaging in safety behaviors, and this exposure will continue until substantive reductions in fear occur. Exposure based treatments facilitate extinction learning such that associations between the initial situation and fear attenuate and novel learning about the true nature of the situation occurs [3,4].

Notwithstanding the considerable body of literature substantiating CBT as a gold standard intervention for anxiety disorders, certain combination strategies may promote greater efficacy [5]. Recent research has attested to the potential utility of neuroenhancers—which has also referred to as cognitive enhancers—as a way to augment adaptive learning that occurs during treatment. Several studies have suggested that administering such neuroenhancers prior to successful exposures might enhance treatment outcome [6,7]. The current review examines the empirical basis of several neuroenhancers, including d-cycloserine, yohimbine, cortisol, catecholamines, oxytocin, modafinil, and selected nutrients.

## 2. D-cycloserine

The modulatory role of the glutamatergic N-Methyl-D-Aspartate (NMDA) receptor in extinction learning has received empirical support from an increasing body of animal literature. Recent research on D-cycloserine (DCS; d-4-amino-3-isoxazolidinone), a partial agonist of the NMDA receptor complex, has fostered interest in the role of glutamatergic transmission in anxiety disorders. DCS has been implicated in the consolidation of new learning during extinction and, thereby, memory enhancement by acting on the NMDA receptors in the amygdala [8,9]. Unlike SSRIs, this agent itself does not produce an anxiolytic effect, but rather enhances successful extinction learning, thereby augmenting the efficacy of exposure therapy [10]. The effects of DCS may be sensitive to contextual conditions, as there is evidence that suggests that the maintenance of extinction learning occurs only in the original learning context [10].

Prior to its recent use in memory enhancement, DCS had been administered as an antibiotic for tuberculosis. The first study to investigate its utility in augmenting exposure therapy assigned height phobic patients to receive either DCS or pill placebo prior to exposure [11]. Results suggested that DCS augmented therapy even with low dosages (i.e., 50 and 250 mg) when administered one hour prior to the exposure session. In fact, patients in the DCS condition showed maintenance of treatment gains post treatment over a three month follow-up period and willingly participated in self-exposure to heights more frequently than did those in the placebo condition. Because patients who received DCS experienced greater reductions in acrophobic symptoms relative to those who received pill placebo, this study provided initial support that DCS may augment memory consolidation of successful exposure experiences [11].

The successful results of this trial encouraged later research to replicate and extend these results across other anxiety disorders. In a randomized, double-blind clinical trial, Hofmann *et al*. [6] examined the comparative efficacy of exposure therapy combined with either 50 mg DCS or pill placebo. In both conditions, patients with social anxiety disorder received the appropriate pill one hour prior to the exposure session for four of the five sessions in the protocol. The results of the current study substantiated those of Ressler and colleagues [11]. Patients who received exposure therapy combined with DCS evidenced significantly greater reduction in social anxiety symptoms at post-treatment and one-month follow-up assessments Otto *et al*. [12] extended this research by examining the DCS augmented exposure for panic disorder. Patients were randomized to receive either DCS or pill placebo prior to three sessions of a five session exposure protocol, in which patients expose themselves to various interoceptive sensations. Compared to patients in the placebo condition, those treated with the combined DCS-exposure intervention achieved better treatment outcomes at both post-treatment and one month follow-up. Specifically, more individuals in the combined condition had clinically significant remission in symptoms (77% vs. 33%).

An important consideration of DCS administration regards its timing. Because peak blood levels of DCS occur within a narrow time window (e.g., four to six hours after ingestion), judicious administration of this pharmacological agent entails matching timing of DCS with timing of extinction learning [13]. Results from our clinical trials suggest that exposure augmentation was accomplished with 50 mg of DCS one hour before each of 5 exposure sessions [6,12,14]. In addition, this administration schedule was associated with in a benign side effect profile, as patients could not distinguish 50 mg of DCS from pill placebo. Thus, extant literature suggests that optimizing DCS requires single, small doses one to two hours prior to exposure at one-week intervals.

Another consideration relevant to the clinical utility of DCS is the success of the exposure. Because it functions as a memory enhancer, DCS augments the learning and reconsolidation of whatever experiences and memories become active during an exposure session. Lee *et al*. [15] have shown that reconsolidation processes dominate during briefer sessions, whereas extinction learning processes dominate during longer sessions. Little extinction occurs if stimulus re-exposure is brief, as the fear memory becomes dominant. This would indicate that administration of DCS would enhance reconsolidation of the fear memory and, thus, result in clinically contraindicated effects [13]. A reanalysis of a recent trial substantiated that administering DCS during insufficient extinction learning (e.g., very brief exposures, inadequate reduction of within session fear levels, etc.) compromises treatment outcome [16,17]. Results indicated that, although CBT with DCS did not outperform CBT with placebo, patients who both received DCS and experienced lower post-session fear levels accomplished better outcome than did those receiving pill placebo. Thus, it appears that several clinical factors must be considered, as exposure success moderates the overall benefit of adjuvant DCS. Future research could extend our knowledge by identifying the conditions under which DCS enhances treatment gains.

## 3. Yohimbine

Yohimbine hydrochloride (YOH) is a purified form of the African yohimbine bark and functions as a selective competitive alpha2-adrenergic receptor antagonist. YOH has received increased attention regarding its clinical utility because of recent studies examining its role as a potential augmentation strategy for extinction-based treatments. YOH increases extracellular norepinephrine by blocking autoreceptor inhibition of norepinephrine release, which could facilitate extinction learning in exposure therapies [18].

Extant research has produced contradictory results regarding the role of YOH on extinction learning. Notwithstanding one early study, which suggested that YOH augmented the rate of extinction learning in rodents, subsequent research has yet to replicate these findings, and some studies have even found YOH to impair animal extinction learning [19]. O’Carroll *et al*. [20] extended this research to human subjects and determined that the administration of 20 mg of YOH prior to observing emotionally provocative images augmented subsequent recall. Furthermore, administering 50 mg of metaprolol (a noradrenaline blocker) in the same study eventuated in worse memory recall, suggesting that YOH’s stimulation of the noradrenergic system facilitated emotional learning.

Despite the paucity of literature examining the YOH in the context of extinction learning, some recent studies have supported its use in clinical settings as an adjunct treatment to exposure therapy. Powers *et al*. [21] conducted a trial in which participants with claustrophobia were randomized to receive either 10.8 mg of YOH or pill placebo one hour prior to two 60-minute sessions of in-vivo exposure therapy consisting of sitting in a closed, dark chamber. Those receiving YOH evidenced more robust reductions in claustrophobia symptoms. A more recent treatment outcome study has replicated the clinical utility of adjuvant YOH for individuals with social anxiety disorder [7]. Patients received either 10.8 mg of YOH or pill placebo prior to each of four exposure sessions. Results suggested that YOH augmentation, relative to placebo augmentation, expedited treatment improvement and entailed lower levels of social anxiety symptoms. Though further research is warranted, these initial studies provide tentative support for the utility of YOH in augmenting exposure therapy. The collective results demonstrate initial promise for YOH’s utility in augmenting exposure therapy.

## 4. Cortisol

Stress activates the hypothalamic pituitary adrenal axis (HPA), which causes the adrenal cortex to release glucocorticoids such as cortisol [22]. The ability of the HPA axis to react to stress is dependent on how well glucocorticoids control the release of adrenocorticotrophic hormone (ACTH) and corticotropin releasing hormone (CRH). Glucocorticoids exert this control by binding to the glucocorticoid receptors (GR) and the mineralocorticoid receptors (MR) located throughout the brain [22]. Specifically, the basolateral complex of the amygdala may play an important role in memory consolidation [23]. If GR agonists are administered to this area of the amygdala post-training in an inhibitory avoidance task, there is an enhanced memory consolidation effect, which is blocked with GR antagonists [24]. In addition to cortisol being released in response to stress, there is a diurnal level of cortisol which is present and follows a circadian rhythm pattern. A high level of cortisol is released in the waking hours of the morning and a low level is released in the evening [25].

Chronic elevations of cortisol have been shown to impair memory, however, short-term increases in cortisol may increase emotional consolidation and extinction learning [22,26]. The relationship between acute stress, glucocorticoid levels and cognition follows the pattern of an inverted U-shaped curve with mid-range doses improving memory consolidation and high or low doses not improving and possibly impairing memory [27,28]. In addition, experimental animal research has found that glucocorticoids impair the reconsolidation of existing memories [29,30] and aid in the consolidation of extinction memory, whereas decreasing or eliminating glucocorticoid function impairs this extinction learning [29,31,32,33]. The enhancing effect of glucocorticoids on memory consolidation has been related only to emotionally arousing experiences and not neutral information [23]. Thus, utilizing cortisol during extinction learning in clinical trials could be very effective since physiological arousal is paramount to effective exposure and extinction.

Research has shown that endogenous and exogenous cortisol levels enhance extinction learning during behavioral exposure treatment for anxiety disorders. Because cortisol follows a circadian rhythm pattern, Lass-Hennemann and Michael [34] examined whether conducting exposure sessions in the early morning compared to the later evening could be enhanced by the endogenous high levels of cortisol in the morning. In fact, they demonstrated that subjects who received the exposure session in the morning showed greater reduction of fear of spiders than those who had the session in the evening. In a second study of specific phobia, Soravia *et al.* [35] administered cortisol or placebo to subjects one hour before exposure to a spider photograph over 6 trials in the span of two weeks. Subjects who received cortisol showed a greater reduction of fear compared to those who received placebo. More recently, these authors [36] replicated the study using two sessions of in vivo exposure to live spiders and found that those subjects who received cortisol reported fewer specific phobia symptoms and less subjective fear and physical distress at the one month follow-up compared to those who received placebo. In a randomized control trial (RCT) of specific phobia of heights, subjects were given cortisol or placebo one hour prior to exposure therapy [37]. The subjects who received the cortisol were significantly less symptomatic during post-treatment and one-month follow-up assessments compared to those who received placebo. This experimental RCT provides strong support for the role of cortisol in enhancing extinction learning for specific phobia. Finally, for subjects who had panic disorder and agoraphobia, higher levels of endogenous cortisol were linked to enhanced extinction learning, which was demonstrated by faster rates of clinical improvement [38]. In another study, the panic disorder subjects with the lowest levels of cortisol during the exposures evidenced the poorest treatment outcome [39]. In summary, this research shows that cortisol can function as an enhancer for exposure therapy for specific phobia and panic disorder possibly by increasing the ability of subjects to retain the newly acquired extinction memory after the exposure trials.

Glucocorticoids have also been shown to enhance encoding of extinction for those with PTSD. In a double blind placebo control trial, Suris *et al.* [40] administered glucocorticoid or placebo after one traumatic memory exposure trial and found that those subjects who received the glucocorticoid after exposure showed decreased numbing and avoidance symptoms of PTSD compared to those who received the placebo. Glucocorticoids have also been shown to decrease the ability to retrieve a previously partially encoded traumatic memory. Aerni *et al.* [41] administered low doses of cortisol daily for one month to three subjects with PTSD and found that they had a significant reduction of intensity related to flashbacks, physiological distress, and nightmares. Taken together, these findings suggest that cortisol may play an important role in the facilitation of new extinction learning and also inhibit retrieval of a previously encoded traumatic memory.

When considering the clinical utility of cortisol in improving treatment outcome, the interfering role of anxiolytic medications in extinction learning should be highlighted. Research shows that anxiolytic medications suppress glucocorticoids [42,43]. This mechanism of cortisol suppression may explain why combining exposure based CBT and anxiolytic medications is not effective [26]. Future clinical trials should measure and statistically control for anxiolytic use when examining the efficacy of cortisol in enhancing extinction learning during exposure. Finally, De Quervain *et al.* [23] state that because glucocorticoids impair memory retrieval and increase new extinction learning for emotional memories, they may be very beneficial in augmenting treatment for anxiety, trauma and stress related disorders.

## 5. Catecholamines

Catecholamines are secreted by the adrenal glands in response to stress. These hormones include dopamine, epinephrine, and norepinephrine. High concentrations of dopamine have been found in the dorsolateral prefrontal cortex (PFC) [44], playing a role in representational or working memory [45]. In nonhuman primates, deficits in dopamine in the dorsolateral PFC produced a decline in working memory [46]. Specifically, injecting a dopamine antagonist at the D1 receptor site in the dorsolateral PFC was related to a response latency and decreased accuracy for a spatial learning task in rhesus monkeys, showing that the dorsolateral PFC and dopamine play an important role in working memory [46]. Research shows that D1 receptor activation in the PFC can positively or negatively affect working memory depending on the level of stimulation. The relationship between cognitive performance and D1 stimulation levels follows an inverted U based curve, suggesting that too much or too little D1 agonist stimulation disrupts performance [47]. In addition, D1 receptor stimulation increases the strength of the mental representations in working memory by shutting off weak inputs on the PFC and strongly stabilizing one or a small set of representations. This allows the organism to pursue one goal over others [47].

Dopamine is also linked to motivation and reward seeking [48], which has implications for substance use disorders. In addition, a high level of dopamine activation is associated with low levels of serotonin or GABA in areas of the brain associated with the pathophysiology of anxiety disorders [49]. Specifically, dopamine receptors that have a higher binding potential in the striatal and mesolimbic areas of the brain are related to an increased risk of obsessive compulsive disorder (OCD) and anxiety disorders [50,51].

Because catecholamines such as epinephrine and norepinephrine are associated with anxiety symptoms, researchers have tried to manipulate the availability of dopamine by using selective serotonin reuptake inhibitors (SSRIs) [52]. SSRIs increase the availability of serotonin in the synapse and this increased serontonin inhibits the release of dopamine. However, for patients with OCD, these medications are not effective alone and research indicates that approximately half of patients are resistant to this psychotropic treatment [52]. Because there is a higher binding potential for dopamine at its receptor sites in the striatal region, future research could be conducted to examine the efficacy of dopamine antagonists to decrease physical symptoms of anxiety in OCD.

## 6. Oxytocin

Oxytocin is a neuropeptide, which plays an important role in social cognition and behavior [53–55]. Social cognition involves psychological processes that underlie the ability of people to take advantage of being part of a social group [56]. Also, social cognition is essential to maintaining interpersonal relationships [57]. Oxytocin is synthesized by neurons in the paraventricular and supraoptic nuclei of the hypothalamus. It predominates in the posterior lobe of the pituitary gland, where it is released into peripheral circulation. It is also released from neuronal dendrites into extracellular space where it can reach local and far-reaching targets. Finally, oxytocinergic neurons project to other areas in the brain such as the amygdala, hippocampus, striatum, suprachiasmatic nucleus, bed nucleus of the stria terminalis and brainstem [58].

Researchers who have examined the relationship between oxytocin and human behavior have measured it in blood plasma to investigate its peripheral actions as a neuropeptide. They have also measured it in cerebrospinal fluid (CSF) to see its central nervous system (CNS) effects as a neuromodulator and neurotransmitter. Research examining oxytocin as a neuropeptide has demonstrated that a higher level of plasma oxytocin is associated with increased trust and trustworthiness [59], increased levels of partner support based on subject ratings [60], and decreased ratings of anxiety and depression [61]. In contrast, low plasma levels of oxytocin have been associated with schizophrenia, autism spectrum disorders and depression compared to normal controls [62,63]. Nevertheless, the picture of the relationship between oxytocin on social behavior and psychopathology is not uniform. Some researchers found that high plasma levels of oxytocin were associated with relationship stress in women [65], and increased levels of social anxiety [66]. Also, it is uncertain whether these findings of plasma levels of oxytocin acting as a neuropeptide mirror the neuromodulatory and neurotransmitter action of oxytocin in the CNS. Future experimental research is needed to examine whether the form of oxytocin administration (i.e., intranasal versus systemic) affects reuptake by the CNS and bloodstream in different ways [67].

In experimental research, oxytocin given to subjects intranasally is related to an increase in prosocial behavior, and improves emotion recognition and social memory [55]. In one seminal study, researchers tested the effect of intranasal oxytocin administration on the willingness of people to exchange money with one other [54]. They found that those subject investors who received oxytocin gave significantly more money to the subject trustees, compared to those who received the placebo. The trust and risk portion of the study referred to the ignorance of the subject investors regarding whether the subject trustees would share their money after the transaction. The overarching results indicate that oxytocin may be related to increased trust in others. In fact, further research shows that subjects who were given oxytocin trusted others more than those in the placebo group and the effect of oxytocin on willingness to take risks was moderated by whether the subject’s interaction was in a social context [68,69]. In another study, researchers found that when a breach of trust was made during a game involving trust, those subjects who received oxytocin still made choices showing that they trusted others on future trials. This relationship was not found for those who received the placebo, who changed their behavior based on the breach of trust [70].

Due to oxytocin’s effect on increasing trust within the social relationship context, researchers have examined whether oxytocin can benefit patients who suffer from disorders involving impairment in social interactions, such as social anxiety disorder, autism spectrum disorders and schizophrenia [71]. In one study, researchers tested whether administering oxytocin as an augmentation to a 5-weeks cognitive behavioral therapy (CBT) exposure treatment for social anxiety disorder would improve treatment outcome [72]. Subjects in the oxytocin group did better on speech performance and appearance compared to those in the placebo group, however, the severity of social anxiety symptoms did not differ between the two groups. The explanation for this null finding on social anxiety symptoms remains unanswered, but one reason could be that the best dose response amount and timing of administration has not been established across multiple research labs. Most studies examining intranasal oxytocin administration on social behavior in humans have used one dose of 24 international units (IU) of oxytocin [73], but it is uncertain as to whether this is the optimal dose for anxiety disorders. To determine whether oxytocin can be used clinically, additional research should be conducted to examine how oxytocin affects fear related circuitry and the best timing and amount of oxytocin to be used to decrease anxiety symptoms.

Research on brain reactivity during an emotional face matching task, has shown that subjects with social anxiety disorder showed heightened reactivity in the amygdala in response to fearful faces compared to healthy control subjects. However, when social anxiety disorder subjects were given oxytocin, the presentation of fearful faces did not evoke the same amygdala reactivity that was present at baseline [74]. In a second study these researchers examined brain reactivity in response to sad faces for subjects with social anxiety disorder. They found that oxytocin decreased hyperactivity in the medial PFC and anterior cingulate cortex (ACC) in response to sad faces for those with social anxiety disorder to the extent that the activation did not significantly differ from the healthy controls [75]. Moreover, it appears that the effect of oxytocin is specific to particular subgroups of individuals [76]. In summary, the research indicates that oxytocin could increase treatment efficacy for patients with social anxiety disorder by decreasing activation of the amygdala, medial PFC and ACC in response to fearful or sad faces.

## 7. Modafinil

Modafinil is a psychostimulant approved by the FDA to treat extreme sleepiness, which is present in shift work, narcolepsy, and obstructive sleep apnea [77]. Modafinil binds to and obstructs the dopamine transporter and norepinephrine transporter, causing significant increases of dopamine [78] and norepinephrine in the extracellular space [79]. In addition research shows that it decreases levels of GABA and increases levels of histamine, orexin, glutamate and serotonin [77]. Also, modafinil has been used off label to treat the cognitive impairment present in psychiatric disorders such as attention deficit hyperactivity disorder, depression, and schizophrenia [77].

Research on modafinil’s effect on memory and cognitive functioning has been conducted on samples of healthy humans, those with psychiatric disorders and animals [80–82]. Modafinil has been shown to improve spatial working memory in rats [84]. The improvement in memory performance is dependent on dose and timing, with stronger effects shown with sustained doses of modafinil [77]. In humans, modafinil exerts its effects by improving working memory, memory recognition, and attention and increasing ability on cognitive control tasks. For patients diagnosed with attention deficit hyperactivity disorder, depression and schizophrenia, modafinil may enhance executive functioning and other PFC functioning [77].

Studies of the relationship between modafinil and anxiety have shown mixed results. In healthy human subjects, modafinil reduced amygdala response to emotionally salient information, such as fearful stimuli [85]. In animal research with monkeys, modafinil was found to heighten nocturnal activity following multiple or single doses, but it did not result in decreased anxiety [80]. In human research the effect of modafinil on anxiety may be contingent on the amount of drug used. Modafinil in doses of 200–800 mg was related to increased anxiety in healthy adults [83]. In a second study researchers found that that 100 mg of modafinil given in one dose was related to a greater level of anxiety compared to placebo, yet, 200 mg of modafinil did not result in an increase in anxiety [82]. In clinical RCTs, repeated doses of modafinil were linked to higher levels of anxiety in patients who had obstructive sleep apnea [86] and patients diagnosed with multiple sclerosis [87]. In summary, modafinil has few side effects in humans but is associated with increased anxiety in healthy and clinical populations, thus, its efficacy as a neuroenhancer for increasing new habituation learning is uncertain.

## 8. Nutrients: Caffeine and Omega-3 Fatty Acids

Several studies have explored the effects of naturally occurring substances such as omega-3 fatty acids and caffeine on anxiety symptoms [88–95]. In a study investigating the effect of the caffeine challenge test on panic induction tasks (e.g., CO_2_ inhalation, breath holding and hyperventilation), results indicated that caffeine influences fear circuitry [93]. Specifically, the unpleasant physical symptoms generated by caffeine consumption were less well tolerated among individuals with panic disorder. Culver *et al.* [93] demonstrated that caffeine administration may enhance relapse prevention after individuals achieve successful extinction learning. Some research has indicated that the extinction effects of caffeine might be state-dependent. When caffeine was administered as an adjunct to exposure therapy for spider phobia [94], those who experienced congruent drug states, (i.e. consuming the same agent (caffeine or pill placebo) at both test and follow-up periods), evidenced reduced return of fear compared with individuals who experienced incongruent drug states, (i.e. consuming different drugs at both time-points).

Omega-3 fatty acids, which compose mammalian brain tissue, can be easily synthesized from foods such as canola, flaxseed, and soy. Constituting approximately 10–20% of brain fatty tissue, docosahexaenoic acid and other forms of fatty acids are not easily synthesized from those foods and are often consumed from natural sources such as fatty fish (e.g., tuna and salmon) [97]. Animal models suggest that insufficient fatty acids are related to neurocognitive deficits, greater levels of aggression, anxiety, and depression. Moreover, human research has indicated that deficits in omega-3 fatty acids are associated with psychopathologies, such as attention deficit hyperactivity disorder [89], depression [98] and schizophrenia [99].

Despite the dearth of evidence supporting the efficacy of omega-3 fatty acid supplements, several studies have investigated them as an adjunct to pharmacological treatment for depression, bipolar disorder, and attention deficit hyperactivity disorder, revealing modest findings [88,89,91,92]. Continued research on omega-3 supplements’ benefits for anxiety treatment could be useful, given the connection between omega-3 deficits and elevated anxiety

## 9. Conclusion

In this review the efficacy of DCS, yohimbine, cortisol, catecholamines, oxytocin, modafinil, and selected nutrients as neuroenhancers for extinction learning for anxiety was examined. Overall, DCS was shown to have the most empirical support, yet the timing of the optimal DCS administration remains uncertain [17]. There is partial evidence that cortisol, catecholamines, yohimbine and oxytocin could also act as neuroenhancers but further research must be conducted to discern the relationship between these agents and enhanced extinction learning. Finally, there was less evidence to support modafinil, nutrients and botanicals as strong neuroenhancers for learning.

Some of these neuroenhancers, especially DCS, are promising for treating anxiety disorders because these agents improve the efficacy of extinction learning, which is integral in exposure treatment for anxiety disorders. They facilitate learning new memories through habituation and extinction, and these safe memories will override the previous fear memories. Also, some of these agents such as DCS target the NMDA receptors in the amygdala, which underlies the pathophysiology and maintenance of anxiety disorders. Thus, utilizing neuroenhancers as an adjunct to CBT represents a promising translational effort, by taking the experimental learning bench research to clinical practice. In contrast, using combination anxiolytic and CBT treatments did not come from a theoretical understanding of the mechanism of action, and has been proven less effective [100]. If the clinical utility of this treatment is demonstrated by multiple labs and research groups, effectiveness research trials can be conducted to disseminate this treatment to community run clinics. Disseminating this treatment to more patients in the real world will decrease the number of patients suffering from the consequences of pathological anxiety (e.g., loss of work, poor job performance, disability, etc.). Further research must be conducted to examine the long-term efficacy of neuroenhancers with CBT for anxiety disorders. Yet, based on short-term follow up findings it is promising. Finally, the tolerability of neuroenhancers in this review is very good with few side effects, which decreases patient burden compared to anxiolytic medications which have more side effects. Future research with human RCTs is needed to test long-term effects of neuroenhancers with CBT and to examine whether these agents can be effectively administered after a treatment session to tailor the drug administration, such that the neuroenhancer is only given after adaptive learning has occurred.
